# Differentiation of Human Embryonic Stem Cells to Regional Specific Neural Precursors in Chemically Defined Medium Conditions

**DOI:** 10.1371/journal.pone.0002122

**Published:** 2008-05-07

**Authors:** Slaven Erceg, Sergio Laínez, Mohammad Ronaghi, Petra Stojkovic, Maria Amparo Pérez-Aragó, Victoria Moreno-Manzano, Rubén Moreno-Palanques, Rosa Planells-Cases, Miodrag Stojkovic

**Affiliations:** 1 Cellular Reprogramming Laboratory, Centro de Investigación Príncipe Felipe (CIPF), Valencia, Spain; 2 Sensorial Biology Laboratory, Centro de Investigación Príncipe Felipe (CIPF), Valencia, Spain; City of Hope Medical Center, United States of America

## Abstract

**Background:**

Human embryonic stem cells (hESC) provide a unique model to study early events in human development. The hESC-derived cells can potentially be used to replace or restore different tissues including neuronal that have been damaged by disease or injury.

**Methodology and Principal Findings:**

The cells of two different hESC lines were converted to neural rosettes using adherent and chemically defined conditions. The progenitor cells were exposed to retinoic acid (RA) or to human recombinant basic fibroblast growth factor (bFGF) in the late phase of the rosette formation. Exposing the progenitor cells to RA suppressed differentiation to rostral forebrain dopamine neural lineage and promoted that of spinal neural tissue including motor neurons. The functional characteristics of these differentiated neuronal precursors under both, rostral (bFGF) and caudalizing (RA) signals were confirmed by patch clamp analysis.

**Conclusions/Significance:**

These findings suggest that our differentiation protocol has the capacity to generate region-specific and electrophysiologically active neurons under *in vitro* conditions without embryoid body formation, co-culture with stromal cells and without presence of cells of mesodermal or endodermal lineages.

## Introduction

Human embryonic stem cells (hESC) are pluripotent cells that can be propagated *in vitro* for a long period and represent a theoretically inexhaustible source of precursor cells that could be differentiated into any cell type to study human development or treat debilitating diseases [1]–[3]. Therefore, derivation of neural progenitors from hESC holds promise to investigate human neurogenesis, to study the development of the central nervous system (CNS) and for potential cell therapy applications to treat Parkinson's disease or spinal cord injury [4].

At this time, hESC differentiation towards neural lineages has several concerns. For instance, the current protocols used to induce neural conversion of hESC include the presence of stromal cell lines (PA6 or MS5), Matrigel or conditioned medium including a multistep procedure which involves formation of embryoid bodies (EBs) [3], [5]–[13]. This bears risks of pathogen cross-transfer or contamination with non-neural cells limiting the efficiency and specificity of the differentiation protocols and future medical application of differentiated hESC. Protocols with EBs yield a small fraction of neural lineage cells due to the presence of cells of mesodermal or endodermal origin. For these reasons, efforts have been initiated to develop feeder-free conditions for growth and neural differentiation of hESC. Manipulation of signalling regulators (bFGF, Wnt, Noggin, and BMP) has facilitated the development of feeder-free conditions for differentiation of hESC towards neural lineages [6], [11]–[15].

Controlled generation of neural progenitors in feeder- and animal-free conditions avoiding the formation of EBs is therefore a desirable approach for further application of those cells in regenerative medicine.

Here we report efficient differentiation of hESC towards very defined neural lineages applying a very simple *in vitro* protocol which includes usage of animal-free components of extracellular matrix (ECM) and chemically defined medium. In addition, this protocol permits controlled differentiation towards regional specific type of neuronal cells by exposing the rosettes to different signalling factors.

## Methods

### Cell Culture

In this study we obtained the same results with both hESC lines. Primary hESC colonies (H9 and H1 lines, WiCell Inc., Madison, WI) were mechanically dispersed into several small clumps, which were cultured on fresh commercially available human foreskin fibroblasts (American Type Culture Collection, Manassas, VA, USA), inactivated by mitomycin C in ES medium containing Knockout-DMEM (Invitrogen), 100 µM ß-mercaptoethanol (Sigma), 1 mM L-glutamine (Invitrogen), 100 mM nonessential amino acids, 20% serum replacement (SR; Invitrogen), 1% penicillin-streptomycin (Invitrogen), and 8 ng/ml basic fibroblast growth factor (bFGF; Invitrogen). ES medium was changed every second day. Human embryonic stem cells were passaged by incubation in 1 mg/ml collagenase IV (animal-free, Invitrogen) for 5–8 minutes at 37°C or mechanically dissociated and then removed to freshly prepared human foreskin fibroblast layer.

### Neural Differentiation

Two to three pieces of domed hESC colonies were transferred to human matrix coated plates composed of 10 µg/cm^2^ human collagen IV (Sigma), 0.2 µg/cm^2^ human vitronectin (Sigma) and 5 µg/cm^2^ human fibronectin (Sigma) in modified TeSR1 medium [16]. Instead of adding human serum albumin to the TeSR1 medium, we used 15.5 ml of Voluven 6% (Fresenius-Kabi) per 100 ml medium.

The day when the cells attached was signed as D0. After the appearance of rosette structures (D2) the cells were maintained for 5 additional days in the same medium. From D7 to D14, the medium was changed to GRM supplemented with 10 µM/ml all-trans-retinoic acid (GRM/RA), or 8 ng/ml human recombinant bFGF (Invitrogen; GRM/bFGF). GRM medium consisted of DMEM:F-12, B27 supplement (Invitrogen), 25 µg/ml human insulin, 6.3 ng/ml progesterone, 10 µg/ml putrescin, 50 ng/ml sodium selenite, 50 µg/ml human holotransferrin (Sigma). At D14, cells were plated on ornithine/laminine coated slides and maintained during 4 weeks or more in the presence of GRM/bFGF.

### RNA Extraction and Reverse Transcription-PCR Analysis

Total RNA was extracted using High Pure RNA isolation Kit according to manufacturer's instructions (Roche Diagnostics). cDNA was synthesized using High Capacity cDNA Archive Kit (Applied Biosystems, CA, USA). Amplification was performed on the cDNA using Taq polymerase (Invitrogen). PCR conditions included a first step of 3 min at 94°C, a second step 35 cycles of 15 sec at 94°C, a 30 sec annealing step at 56°C, 45 sec at 72°C, and a final step of 8 min at 72°C. Glyceraldehyde-3-phosphate dehydrogenase (GAPDH) was used as a control gene to evaluate and compare the quality and quantity of different cDNA transcripts. Primer sequences are provided (Table S1).

### Immunocytochemistry

At D28 or D42 the cells plated on polyornithine/laminin-coated permanox slides were washed in PBS and fixed with 4% paraformaldehyde in PBS for 10 minutes. Fixed cells were washed twice with PBS before staining. After permeabilization in 2% Triton and blocking in 1% bovine serum albumin (BSA) for 30 minutes, primary antibodies were applied in blocking buffer for 2 hours at room temperature. The cells were washed three times in blocking buffer before secondary antibody application. Secondary antibodies of goat anti-mouse Alexa-conjugated, goat anti-rabbit Alexa-conjugated (Molecular Probes) were diluted at 1∶500 in blocking buffer and applied to cells for 1 hour at room temperature. After two washes in PBS, mounting medium containing 4′,6′-diamidino-2-phenylindole was applied for nuclear staining. Cells were observed under the fluorescence microscope (Axiowert). For negative controls, primary antibodies were omitted and the same staining procedure was followed. Primary antibody dilutions used included the following: rabbit anti-Nestin (1∶200, Chemicon, Millipore), rabbit anti-Musashi 1 (1∶250; Chemicon, Millipore), mouse anti-beta III tubulin-Tuj1 (1∶300; Abcam), rabbit anti-GFAP (1: 400; Chemicon, Millipore), mouse anti-O4 (1∶100; Chemicon, Millipore), mouse anti-A2B5 (1∶100, Chemicon, Millipore), rabbit anti-GABA (1∶1000, Sigma), rabbit anti-pax6 (1∶250, Chemicon, Millipore), rabbit anti-sox1 (1∶500, Sigma), rabbit anti-serotonin (1∶250, Sigma), rabbit anti-glutamate (1∶1000, Sigma), rabbit anti-HB9 (1∶500, Abcam), mouse anti-Choline Acetyltransferase (1∶500, Chemicon, Millipore), rabbit anti-tyrosine hydroxylase (1∶1000, Chemicon, Millipore), rabbit anti- Gbx1(1∶500,Chemicon), rabbit anti-otx2 (Chemicon), mouse anti-RC2 (DSHB), rabbit anti-BLBP (Abcam). Double staining for Tuj1 and GFAP was used to determine the ratio of neuronal and glial cells. Counting of the cells was done as previously described [14].

### Electrophysiology

All reagents were from Sigma unless otherwise stated. Whole cell recordings used an EPC-10 amplifier (HEKA GmbH, Germany). In all experiments the holding potential was –70 mV. The bath solution consisted of (in mM): 140 NaCl; 4 KCl; 2 CaCl_2_; 10 HEPES; 5 Glucose; 20 Mannitol, pH 7.4. The electrode solution contained (in mM): 144 KCl; 2 MgCl_2_; 10 HEPES; 5 EGTA, pH 7.2. The pipette resistance was 1.5–2 MΩ. Series resistance was compensated for (>70%) and leak substraction performed. Data were low pass-filtered at 3.3 kHz and sampled at 10 kHz. Applied pulse protocols and analysis were carried by Pulse software (HEKA). Action potentials were evoked by applying a variable pulse of current (20–100 pA) to neurons in current-clamp configuration.

In voltage clamp configuration, squared pulse depolarization voltage steps were applied from –30 mV or to +60 mV from the holding potential of −70 mV. Tetrodotoxin (TTX) (Tocris Laboratories) was used to block TTX-sensitive voltage-dependent Na^+^ channels. Replacement of K^+^ by Cs^+^ in the internal solution, or external application of 4-aminopyridine (4-AP) and Tetraethylammonium (TEA) blocked outward K^+^ currents. Receptor agonists glutamate, γ-aminobutyric acid (GABA), dopamine or acetylcholine were tested. The antagonists bicuculline (Tocris Laboratories), 6-cyano-7-nitroquinoxaline (CNQX) and 2-amino-5-phosphonopentanoic acid (AP-V) were examined on neurotransmitter-mediated currents. Data were averaged and represented as means±S.E.M.

## Results

### Controlled Differentiation of hESC on ECM in Chemically Defined Medium

**Figure 1:**
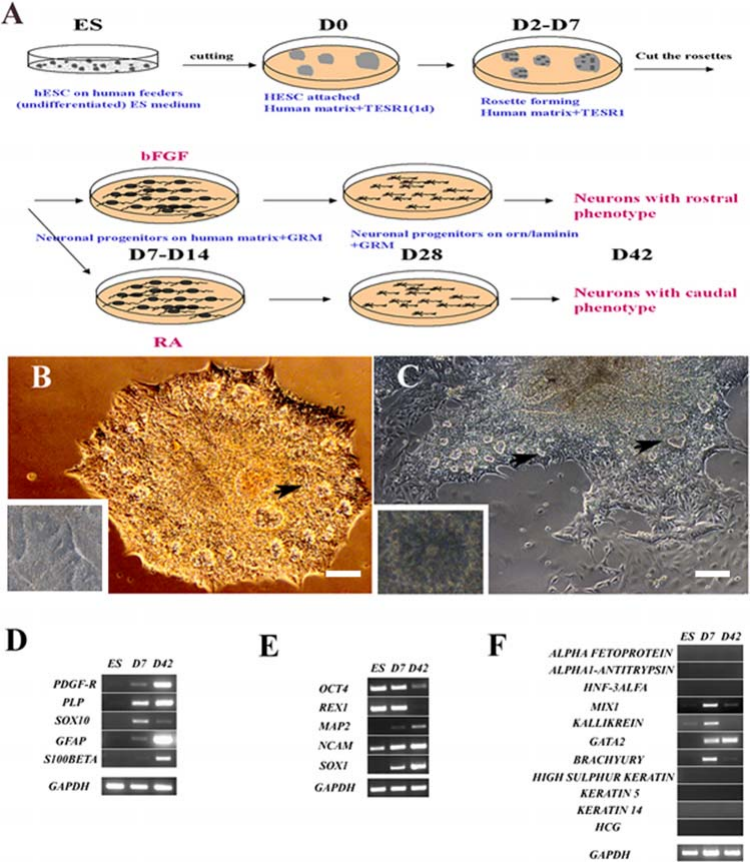
Differentiation of hESC to neural progenitors. (A) Schematic representation of the different steps in feeder-free and chemically defined medium conditions (see Material and Methods). (B and C) After two days in adherent animal-free substrate and modified TESR1 medium the cells were organized into rosettes (indicated with black arrows, also inserts, B and C). (D–F) RT-PCR analysis indicates changes in gene expression of pluripotency and neural markers through three steps of differentiation protocols: (D) oligodendrocyte and astrocytes expression profile; (E) Changes in gene expression of main pluripotency markers and general neural markers. (F) Semiquantitative RT-PCR of some endodermal, mesodermal, epidermal, and trophoblast markers. Bars: (B) 100 µm; (C) 200 µm.

**Figure 2:**
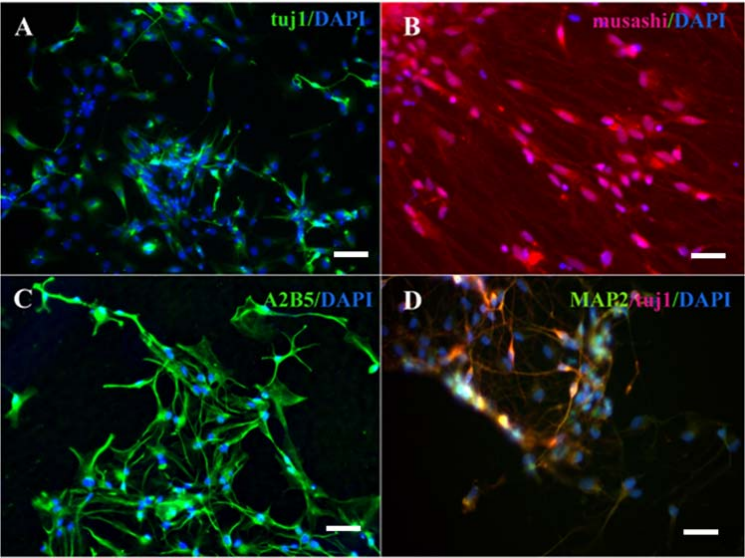
Immunocytochemical characterizations of hESC-derived neuronal precursors at D28. The cells are positive for neural progenitor markers, such as (A) Tuj1 (green), (B) Musashi (red), (C) A2B5 (green), (D) MAP2 (red coexpressed with Tuj1). Blue indicates DAPI. Bars: (A–C) 50 µm; (D) 25 µm.

**Figure 3:**
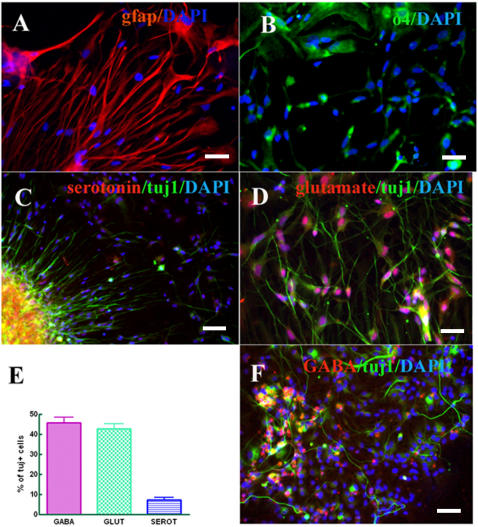
hESC derived neural progenitors give rise to (A) astrocytes (GFAP red) and (B) oligodendrocytes (O4, green). The cells are serotonin^+^ (C; red coexpressed with Tuj1), glutamate^+^ (D; red, coexpressed with Tuj1), and GABA^+^ (F; red, coexpressed with Tuj1). Blue indicates DAPI. GFAP^+^ and O4^+^ cells are stained at D56. Bars: (A, B, D) 25 µm; (C, F) 50 µm. (E) Data were averaged and represented as means±S.E.M.

Undifferentiated hESC (H1 and H9 lines) were maintained on human foreskin fibroblast cells. For the controlled differentiation, pieces from domed colonies were passaged at D0 to the plates previously coated with human collagen IV, fibronectin, vitronectin in PBS in the chemically defined medium. A modified protocol previously described by Ludwig and collaborators [16] of chemically defined hESC medium was applied. After 24 hours the cells attached and revealed typical hESC morphology. At D2, the first sign of neural differentiation emerged with the typical neuroepithelial structures or rosettes (Figure 1B and Figure 1C), and at D5-D7 the cells organized into neural tube-like rosettes with lumens (Figure 1C). Immunocytochemical analyses at D7 revealed that the columnar cells in rosettes, but not the flat, surrounding cells, were positive for *PAX6* (data not shown). Cells at all stages (D2–D7) expressed Nestin, a common neural progenitor marker (not shown). The medium was then replaced at D7 to GRM medium (see Methods) supplemented with basic fibroblast growth factor (GRM/bFGF) for another 7 days (see the scheme in Figure 1A). After 2 days the rosettes started to expand rapidly. To evaluate the neural differentiation potential of cells obtained in our protocol, the rosettes were mechanically cut and plated on ornithin/human laminin coated chamber slides in GRM/bFGF medium. Rosette colonies expanded rapidly on this matrix, adopting at an early stage a spindle-shape morphology, reminiscent of the radial glia [12], [17]. At D14 the vast majority of these cells were Nestin^+^ (89±2%), RC2^+^ (99±1%), Pax6^+^ (87±3%), and BLBP^+^ (95±2%) (Figure S1E). After one day, Tuj1^+^ neurons began to elongate (Figure S1A) and their number increased during further differentiation (Figure S2E). At D28 the cells were positive for the following neuroectodermal markers: Nestin (data not shown), Musashi-1 (Figure 2B), A2B5 (Figure 2C) and MAP2 (Figure 2D). Immunocytochemistry analysis during D14-D21 revealed that the cells were Tuj1^+^ (20% of total cells; Figure 2A) reaching 62% of total cells at D42 (61.8±3.1%; n = 4) of which 46% (45.7±2.7%; n = 3) (Figure 3E) were GABA^+^ (Figure 3F), 43% (42.8±2.4%; n = 4) glutamate^+^ (Figure 3D) and 7% (7.2±1.2%; n = 4) serotonin^+^ cells (Figure 3C). At this stage, 14% of cells were immunoreactive to astrocytes glial fibrillary acidic protein (GFAP) (Figure 3A) and 23% to early oligodendrocytes O4 marker (Figure 3B). During prolonged maintenance, the percentage of neurons progressively decreased with concomitant increase of glial cells percentage (Figure S2).

RT-PCR analyses revealed changes in expression of the common hESC and neural markers during ES, D7 and D42 stages. Specifically, a gradual strong down-regulation of pluripotency markers *OCT4* and *REX1* was observed, accompanied by up-regulation of human neuronal markers *PAX6*, *SOX1*, *NCAM* and *MAP2* (Figure 1E). The downregulation of early (*SOX10)* and upregulation of late precursor markers *PDGF-R* [9], [18] and *PLP* reveals the presence of mature oligodendrocyte cells (Figure 1D). The levels of *GFAP* and *S100β*, markers of astrocyte lineage, were also enhanced, which correlates with immunostaining results. However, semi-quantitative RT-PCR analysis revealed that at D42 the expression of endodermal (*α-FETOPROTEIN, α1-ANTITRYPSIN, HNF-3α*), mesodermal (*MIX, BRACHYURY* and *KALLIKREIN),* epidermal (*KERATIN 5, KERATIN 14, HIGH-SULPHUR KERATIN)* and trophoblast *(HCG)* was completely suppressed (Figure 1F). Only mesodermal marker GATA2 was upregulated, but previous studies showed that this marker is also expressed early and abundantly in the nervous system [19] providing more evidence for the neural profile of our cells.

### Positional Identity and Specification of the Mature Neuronal Population

**Figure 4:**
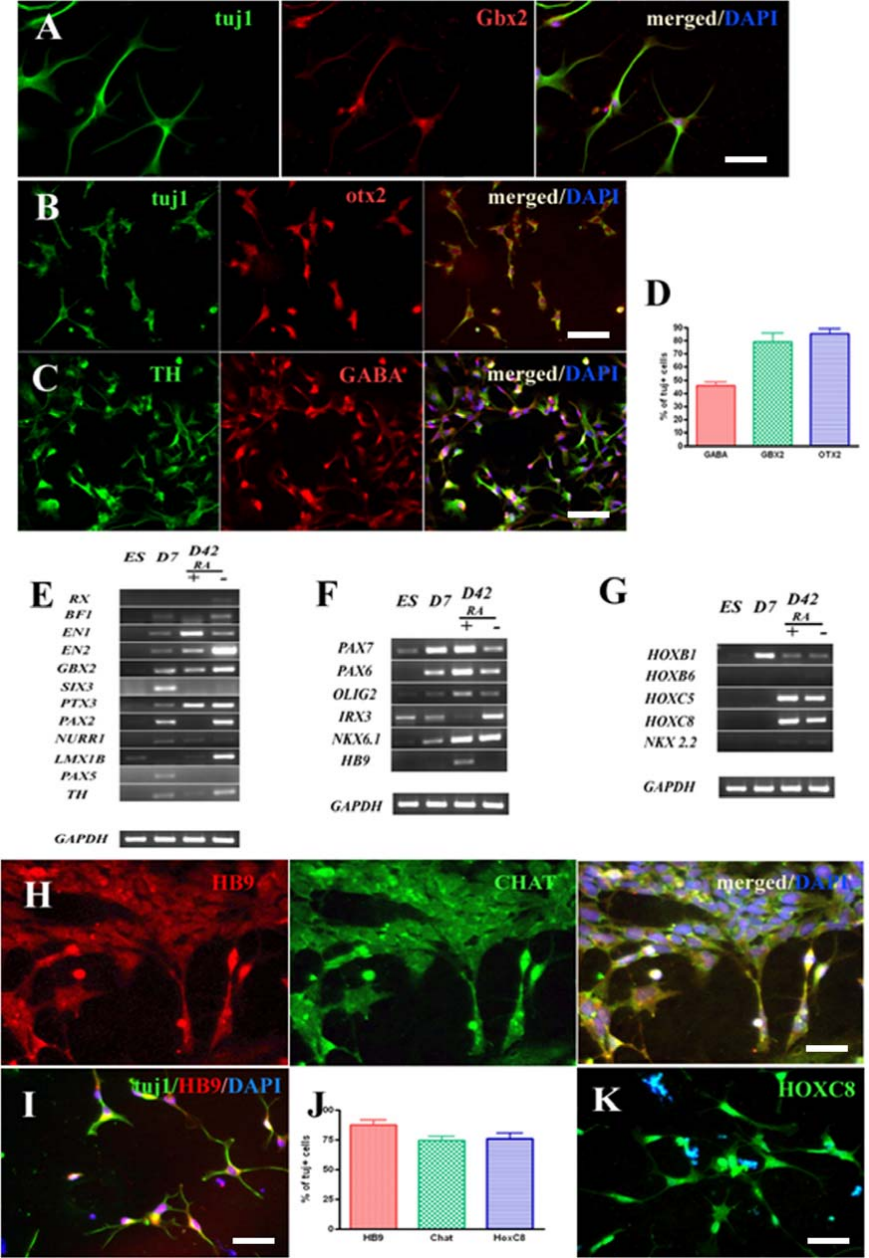
Neuronal specification of cells obtained using protocols with or without RA. The hESC derived neural progenitors display rostral phenotype: (A) the cells are Gbx2^+^ (red, coexpressed with Tuj1) and (B) OTX2^+^ (red, coexpressed with Tuj1). (C) Almost all GABA^+^ cells (red) are TH^+^ (green). (D) The percentage of the cells positive for rostral markers. (E) RT-PCR analysis of rostral markers of hESC-derived neural progenitors with or without RA. (F and G) RT-PCR analysis of spinal cord markers. The hESC derived neural progenitors treated with RA display spinal cord phenotype; (H) almost all HB9 cells (red) are cholinergic (green). (I) The cells are mostly HB9^+^ (red) and Tuj1^+^ (green) positive. (J) Percentage of HB9^+^, Chat^+^, and HOXC8^+^ cells. (K) The cells are also HOXC8^+^ (green). Blue indicates DAPI. Bars: (A) 50 µm; (B, C, H, I, K) 100 µm. (D, J) Data were averaged and represented as means±S.E.M.

To specify the neuronal population types obtained, we first determined the expression of rostral-caudal CNS markers using the RT-PCR method. Almost all genes of rostral-caudal identity initially were expressed at D7. Interestingly, at D42 the cells expressed the ventral diencephalon marker *RX*, telencephalic marker *BF1* [20], [21], as well as the hindbrain marker *GBX2* (Figure 4E). To assess the dopaminergic profile of the generated cells, we analysed transcription factors *PAX2* [22], [23], *PTX3*, *LMX1b* [24]–[27] involved in dopaminergic differentiation finding them upregulated. In contrast, we observed very weak expression of transcripts *EN1*, characteristic for midbrain cells [28] and *NURR1* that has a role in the differentiation of midbrain precursors into dopamine neurons [29]–[32], revealing possible forebrain character of generated cells. Finally, one of the markers associated with the mature dopaminergic neuronal phenotype, tyrosine hydroxylase (TH) was also expressed (Figure 4E).

Immunocytochemistry analysis showed further evidence of dopaminergic phenotype of these neurons. All Tuj1^+^ progenitors were also GABA^+^ (Figure 3D) (46%; 45.7±2.7%; n = 3) and *GBX2* ^+^ (Figures 4A and 4D) (79%; 79.2±6.5%; n = 5), but negative for motoneuron progenitors (*HB9^−^*, *Lim^−^,* data not shown). The majority of GABA^+^ cells (93%) coexpressed TH (Figure 4C). The total population of the Tuj1^+^ cells contained 2% of dopamine positive cells (data not shown), indicating an immature dopaminergic phenotype of these neurons. The RT-PCR analysis did not reveal expression of sensory neuron specific markers (Figure S3). However, the TH^+^ cells were positive for *OTX2* (Figure 4B) (85% of the Tuj1^+^ cells; 85.4±3.6%; n = 5), a transcription factor expressed by forebrain cells [20], suggesting a forebrain identity of the *in vitro*-generated neuroepithelial cells.

To determine whether the neural progenitors have strictly rostral character we next examined the expression of caudal markers (Figures 4F and 4G) such as class I (*IRX3, PAX6, PAX7)* and class II (*OLIG2*, *NKX2.2*, *NKX6.1*), homeodomein proteins important for motoneuron differentiation [33], [34] and found them highly expressed at D7 and D42. In addition, we observed coexpression of some genes involved in dorsal-ventral identity of neural population. Neuronal cells expressed homeodomain proteins *HOXC8* and *HOXC5*, markers expressed in thoracic regions, as well as the weak expression of spinal cord marker *HOXB1* and *HOXB6* (Figure 4G). This profile of neural *HOXC* expression is indicative of spinal cord cells with a rostral cervical identity [35]. This suggests that our protocol, employing bFGF (D7–D14), efficiently supported differentiation towards rostral cells, as well as activation of caudal markers but not generation of cells similar to the ventral most region of the neural tube.

**Figure 5:**
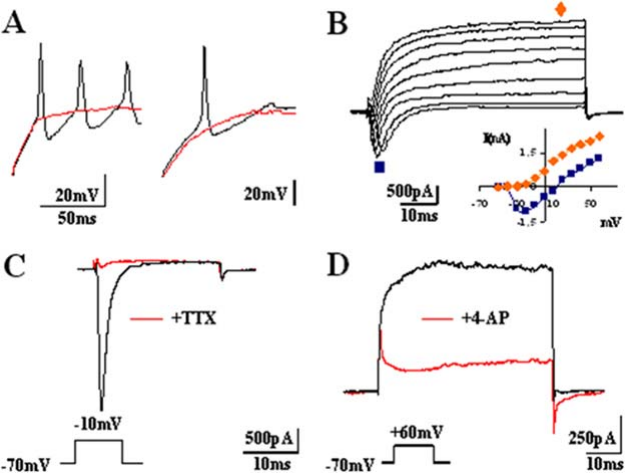
Neuronal excitability and study of voltage-dependent channels in the newly generated neurons by bFGF and RA treatment. (A) Action potentials evoked by depolarizing current steps (40 pA) in two different neurons resulted in different firing patterns. In both cases, the spikes were fully blocked with 1 µM TTX (red). (B) Family of voltage-dependent currents obtained from a –30 mV to +50 mV in 10 mV increasing steps protocol from a V_h_ = −70 mV. An early inward current suggests the presence of voltage-dependent sodium channels, while the second outward component is consistent with the presence of voltage-dependent K^+^ channels, as seen in the I-V relation. (C) Blockade of TTX-sensitive voltage-dependent Na^+^ channel. The early component present in (B) was fully blocked with 1 µM TTX (red trace) (n = 39). (D) The outward current was blocked by 2 mM 4-AP (red trace) (n = 10).

We attempted to alter this rostral-caudal identity of neuronal progenitors by exposing the cells to RA, a well known caudalizing signal, during D7–D14 (Figure 1A). At D28 we observed the same expression profile of neural progenitors compared to the bFGF protocol (data not shown). The neural progenitors acquired a spinal positional identity because the RA treatment completely suppressed or significantly downregulated the rostral markers *RX, BF1, GBX2* as well as midbrain dopaminergic markers *PTX3*, *PAX2*, *NURR1, LMX1B,* and *TH* (Figures 4E and 4F). The transcript *EN1* was fully expressed (while *EN2* was downregulated). In contrast, the caudal markers were strongly upregulated or remained unchanged. Importantly, upon exposure to RA, transcript *IRX3* was suppressed and *OLIG2* was upregulated indicating that RA has influence in dorsoventral neuronal identity *in vitro*. Interestingly, coordinate expression of all these factors promotes the expression of *HB9*, the common marker of motoneurons. Immunocytological analysis at D42 showed that Tuj1^+^ cells contained 87% of HB9^+^ cells (87.3±4.1%; n = 4; Figures 4E and 4J), 74% Chat^+^ (74.5±3.3%; n = 4; Figure 5H, presented in coexpression with HB9), and 76% HoxC8^+^ cells (76.3±4.4%; n = 4; Figure 4K). These results indicate that hESC, initially differentiated under chemically defined conditions to neural cells of forebrain-like identity, can be caudalized to a motoneuron identity upon exposure to RA.

### Functional Properties of Neurons Derived from Mature Neuronal Progenitors

The functional characteristics of these differentiated neuronal precursors under both rostral (+bFGF) or caudalizing (+RA) signals were evaluated by patch clamp analysis at D28 to D42 in culture. Cells visually scored as neurons were subjected to conventional standard patch-clamp recordings. To test their ability to fire action potentials, cells were injected with 20-100 pA depolarizing current from a holding potential of −70 mV. As shown in Figure 5A, at D42 79% (n = 117) of tested cells evoked at least one overshooting action potential in GRM/bFGF, while repetitive firing was found in 9% of cells. Interestingly, 100% (n = 57) of cells elicited action potentials in GRM/RA, from which 18% showed multiple spikes. The Na^+^ channel blocker TTX at 1 µM applied to the bath reversibly blocked the action potentials (Figure 5A).

The presence of voltage-gated Na^+^- and K^+^- channels was studied in the voltage-clamp configuration. In the presence of intracellular K^+^, an early and fast transient inward current was followed by a sustained outward current component upon 20 ms voltage-step pulses from −30 to +50 mV in 10 mV steps increments from a V_h_ = −70 mV (Figure 5B). The current-to-voltage relations obtained for the inward and outward current components were characteristic for Na^+^ and K^+^ currents, respectively. This finding was further supported by separately studying those components and blocking them with specific antagonists. For Na^+^ currents, a depolarising step to −10 mV from the V_h_ resulted in an inward current that was totally blocked with TTX (Figure 5C). For K^+^ currents, the outward component evoked at +60 mV from V_h_ was partially blocked (∼70%) with 2 mM 4-AP (Figure 5D) and ∼85% with 10 mM tetraethylammonium (TEA) externally applied (data non shown).

**Figure 6:**
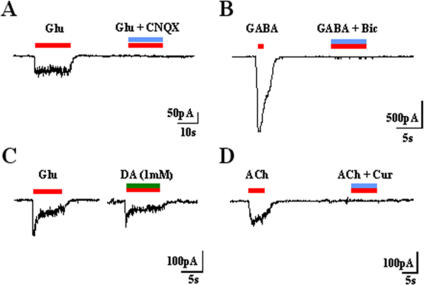
Neurotransmitter sensitivity. (A) Response to 100 µM glutamate (Glu) and blockade of the current by the simultaneous application of the antagonist CNQX (n = 11). The horizontal bar indicates the duration of agonist (red line) and/or antagonist (blue line) or modulator (green line) application. (B): Response of cells to 100 µM of GABA and blocking of the current with the specific GABA_A_ receptor antagonist bicuculline (Bic) (n = 25). (C): Reversible downregulation of Glu currents in cells treated with bFGF by applying 1 mM of the metabotropic neurotransmitter dopamine (DA) for two minutes to the cells (n = 8). (D): Response of cells treated with RA to 100 µM acetylcholine (Ach) (n = 8). The current was fully blocked with 100 µM of (−) tubocurarine (Cur) (n = 6).

We then tested the neurotransmitter sensitivity to GABA, glutamate, dopamine and acetylcholine of neurons present in the cultures. As shown in Figure 6B, the majority of the tested cells (77) evoked whole-cell current of 1267±115 pA (n = 53) when 100 µM of GABA was applied in cells differentiated in GRM/bFGF, similar percentage of GABAergic neurons (81%) resulted in GRM/RA conditions, 870±144 pA (n = 40). These currents were fully blocked by the specific GABAA receptor blocker bicuculline (Figure 6B). Glutamate 100 µM evoked small inward currents (55±6 pA) in ∼60% of tested cells for both types of exposed factors (Figure 6A), which were blocked by 100 µM CNQX but not by APV, thus demonstrating the contribution of non-NMDA receptors to this current.

To assess current modulation by dopamine in these neurons, their differential effect on glutamatergic receptors was assayed. As reported for mammalian neostriatum [35], 1 mM dopamine reversibly downregulated glutamate-evoked currents by ∼47% in GRM/bFGF medium (Figure 6C). Interestingly, in GRM/RA conditions, cells yielded inward currents of 55±13 pA sensitive to acetylcholine (Ach) in ∼40% of tested cells. These currents were fully blocked by the specific blocker (−) tubocurarine, Figure 6D).

## Discussion

We have demonstrated that neural progenitors can be efficiently generated in a one step approach using feeder-free and chemically defined conditions but without formation of EBs. Exposure of hESC to different human ECM substrates leads to the generation of highly enriched neural progenitors without presence of other cells with mesodermal or endodermal characteristics. We also demonstrated that it is achievable to induce both, dorsalmost and ventralmost CNS differentiation of the neuronal progenitors by exposing the neuroectodermal cells to a combination of different exogenous factors.

Our controlled protocol reveals certain advantages over a number of different protocols [1], [4], [7], [8], [11]–[13], [36], [37] already existing describing the generation of neural progenitors. Most of these protocols were based either on spontaneous differentiation of the hESC into a mixture of various cell types [5], [36] or generation of neural progenitors involving cell aggregation or EB formation. Our method includes the initial differentiation of hESC in chemically defined medium and adherent substrate resulting in morphological changes, including rosettes and neural tube-like structures previously identified as typical neural progenitor cells [3], [13], [36]. The data that rosettes express multiple early neural cell markers *SOX1*, *PAX6* and *NESTIN* and efficiently differentiate into neurons and glia cells suggests that the neural rosette cells obtained in our protocol are multipotential neural progenitors with similar characteristic to those obtained with methods including EBs [36]. At the same time we found that expression of pluripotency markers gradually decreased with the onset of expression of early neural markers. After plating, the rosette derived progenitors gave rise to an outgrowth of highly enriched bipolar cells expressing *TUJ1*, *MUSASHI*, *NESTIN*, *A2B5*, and *MAP2* markers typically present in hESC-derived neural progenitors [14]. The yield of neural progenitors was the same or even higher than in previously published protocols where chemically defined medium and adherent conditions were used [11]–[13], [38]. However, these studies did not demonstrate the differentiation towards cells with more specific regional neuronal phenotype. Neural progenitors obtained with our protocol represented three major neural lineages: neurons, astrocytes, and oligodendrocytes similar to previously described methods [5], [9], [15]. Long-term growth of the cells using the same conditions increased the percentage of neurons. Even a gradual shift from neuronal to glial fate was observed. The percentage of oligodendrocytes was 23% at D42, which is similar to efficiency observed in EBs protocols [11], [15]. We showed that neuronal progenitor subtypes give rise to differentiated neurons that generate overshooting action potentials in response to depolarising current injection. Further characterisation of voltage-gated channels revealed the presence of Na^+^- and K^+^-channels as physiological markers of differentiation. Furthermore, K^+^- currents partially blocked by 4-AP appear in both immature as well as mature neurons, however the blockade sensitivity by TEA has been previously found in developing neurons [39]. Moreover, the majority of neuron-like cells responded to GABA and to a lesser extent to glutamate, which has been previously reported in newly generated neurons in developing and adult animals [40]–[42]. We further characterized the positional specification of neurons obtained in our protocol demonstrating controlled differentiation of those cells into specific classes of CNS neurons. Several previous studies showed that it is possible to generate specific neurons from ESC using different methods (mostly using EBs or coculture) [1], [4], [7], [8], [13], [36], [37] and using various concentrations of soluble growth factors [36], [43]. The mature neurons in our protocol acquired mostly rostral character which corroborates with previous co-culture studies showing that the majority of neurons generated express rostral neural markers [44], [45]. In comparison to these studies the efficiency of our protocol was only higher in obtaining forebrain dopaminergic neurons. During the CNS development, regardless of their final position identity, neural plate cells initially possess rostral character [45]. Mature neural progenitors weakly express telencephalic markers, but strong expression of *BF1*, *EN2* and *OTX2* but not *EN1* reveals their forebrain character. Neuroepithelial cells generated in EBs protocols, using chemically defined medium with bFGF, also display forebrain characteristics [36], [43]. Our results corroborate with previous studies [46], [47] that primitive neuroepithelia cells obtained under serum-free and EBs-like conditions treated only with bFGF display rostral phenotype. For instance, Watanabe et al. [48] generated telencephalic precursors from mouse ESC with rostral phenotype using EBs formation, but the percentage of these neurons was much lower than in our study.

Several factors can stimulate dopaminergic neuron development. Nurr1, Lmx1b, and Ptx3 transcription factors are mesencephalon specific, whereas the messenger molecules SHH and FGF8 seem to promote the dopaminergic phenotype irrespective of brain region [49]–[51]. Our results clearly demonstrated that the main dopaminergic markers were strongly upregulated at D42 when we exposed the neuroectodermal cells only to bFGF. The dopaminergic nature of obtained cells was confirmed by electrophysiological recordings. Indeed, dopaminergic neurons in our protocol revealed a modulatory effect of DA on glutamate receptors as previously shown in mammalian neostriatum [52]. It was already reported that co-culture of nonhuman primate ESC with the mouse bone marrow mesenchymal PA6 cells (SDIA) results in TH^+^ midbrain neuronal cell lineage expressing *NURR1* and *LMX1B* genes [8], [44], [53]. Perrier and collaborators [54] showed that hESC can also differentiate into dopamine *Pax2* ^+^ neurons when co-cultured with mouse bone marrow mesenchyme cells which is similar to our feeder-free protocol. The role of bFGF in neural fate determination could be explained by the expression of some Hox genes which are involved in spinal cord differentiation. It has been shown that in response to FGF signaling, cells of prospective caudal neural plate are initially specified either as cells of rostral hindbrain or as Hoxb4^+^/b8^+^/c9^+^ cells which are typical for caudal/thoracic/spinal region [55]. Absence of sensory and mature dopamine neurons suggests that further application of more specific induction factors (for instance, FGF8 and SHH) could improve the targeted differentiation of hESC to midbrain dopaminergic neurons.

We have examined whether the use of extracellular inductive signals, more specifically RA, permits efficient differentiation of hESC into specific classes of CNS neurons. It is well established that RA and FGF exert opposing actions during rostrocaudal regional identity determination of hindbrain and spinal cord progenitor cells [56]. The GRM/RA protocol produced downregulation of rostral transcription factors, especially of genes associated with the dopaminergic phenotype, and upregulation of caudal genes resembling normal patterning of RA in the rostrocaudal axis of the embryonic neural tube. In early somite stages, RA supplied by the paraxial mesoderm and newly formed somites, promotes the expression of Hox genes characteristic of the rostral levels of the spinal cord [56]–[58]. Likewise, rosette cells exposed to RA acquired this spinal positional phenotype as shown in expression profile of all observed caudal genes. A ventralizing effect of RA was also observed by upregulation of *OLIG2* expression, probably at the expense of *IRX3*, in agreement with previous findings suggesting that *OLIG2* suppresses *IRX3* [59] and interacts with progenitor homeodomain proteins, thus activating motor neuron differentiation pathway and *HB9* gene. Indeed, high expression of HB9 and Chat, and functional expression of cholinergic receptors revealed the spinal motoneuron phenotype of obtained neurons. This demonstrates that our differentiation protocol is an improvement, when compared with the protocols where stromal cells [60] or EBs [36], [60], [61] were used. Previous study [62] demonstrated that lysophosphatidic acid inhibits neurosphere formation and the differentiation of neural stem cells derived from hESC through the activation of the Rho/ROCK and the PI3K/Akt pathways. Therefore, further studies are required to conclusively address the expression of region-specific markers, signaling pathways, and to determine whether developing neural processes could be completely mimicked using hESC and *in vitro* defined systems.

In this study, we showed that highly enriched neural progenitor cells, particularly neurons, can be derived efficiently from hESC in a feeder-free and in a chemically defined system. We have shown that after RA treatment neural differentiation is controllable, especially when the neurons acquire different regional position identity. The results reported here provide a very simple and efficient protocol that can serve as an experimental tool to study specific factors and processes involved in human neural development under defined *in vitro* conditions. This is very important for the studies of developmental biology, disease modelling, drug development and cell transplantation.

## Supporting Information

Table S1 (0.12 MB DOC)

Figure S1 Differentiated cells with radial glia phenotype at D14. (A) The cells are Nestin+ (red coexpressed with Tuj1 and RC2, B). Almost all cells were BLBP+ (red coexpressed with RC2, C and PAX6, D). (E) Percentage of Nestin+, RC2+, PAX6+, and BLBP+ cells. Blue indicates DAPI. Bars: (A–D) 50 µm. Data were averaged and represented as means±S.E.M (E).
(10.02 MB TIF)

Figure S2 Time-course of gradual neuronal (Tuj1+) to glial (GFAP+) shift. Images of different time-points: D14 (A), D42 (B), D56 (C), D112 (D). The percentage of the cells was analysed by double immunostaining at different time-points of GRM/bFGF or GRM/RA protocols (E). Bars: (A, B) 50 µm; (C, D) 25 µm. Data were averaged and represented as means±S.E.M (E).
(10.21 MB TIF)

Figure S3 RT-PCR analysis indicates absence of sensory neuron markers. The cells are TH positive, but negative for sensory markers. ES: embryonic stem cells; D7 and D42: day 7 and day 42, respectively.
(0.29 MB TIF)
