# Supplementary material for: Differentiation of Human Embryonic Stem Cells to Regional Specific Neural Precursors in Chemically Defined Medium Conditions
Source: PLoS One. 2008 May 7;3(5):e2122. doi: 10.1371/journal.pone.0002122 (PMC2346555; doi:10.1371/journal.pone.0002122)
Supplement: Table S1 — (0.12 MB DOC) [file pone.0002122.s001.doc]

Supplementary Table 1: Primer sequences of markers used for RT-PCR analysis

| ***Primer*** | Sequence | Size (bp) |
| --- | --- | --- |
| ***OCT4*** | 5´-AGTGAGAGGCAACCTGGAGA-3´ | **273** |
|  | 5´-GTGAAGTGAGGGCTCCCATA-3´ |  |
| ***RX*** | 5´-CTGAAAGCCAAGGAGCACATC-3´ | **409** |
|  | 5´-CTCCTGGGAATGGCCAAGTTT-3´ |  |
| ***SIX3*** | 5´-CAAGTCCACACACACTCCCAC-3´ | **254** |
|  | 5´-CGTCATGCAGGTGGGGTGC-3´ |  |
| ***TH*** | 5´-GTCCCCTGGTTCCCAAGAAAAGT-3´ | **331** |
|  | 5´-TCCAGCTGGGGGATATTGTCTTC-3´ |  |
| PAX5 | 5´-CCGAGCAGACCACAGAGTATTCA-3´ | **403** |
|  | 5´-CAGTGACGGTCATAGGCAGTGG-3´ |  |
| ***SOX1*** | 5´-CAATGCGGGGAGGAGAAGTC-3´ | **464** |
|  | 5´-CTCTGGACCAAACTGTGGCG-3 |  |
| ***OLIG2*** | 5´-AAGGAGGCAGTGGCTTCAAGTC-3´ | **315** |
|  | 5´-CGCTCACCAGTCGCTTCATC-3´ |  |
| ***S100β*** | 5´-GAAGGGGTGAGACAAGGAA-3´ | **310** |
|  | 5´-GTTTGGCTGCTTTCTAATCTC-3´ |  |
| ***EN1*** | 5´-CTGGGTGTACTGCACACGTTAT-3´ | **357** |
|  | 5´-TACTCGCTCTCGTCTTTGTCCT-3´ |  |
| ***EN2*** | 5´-GTGGGTCTACTGTACGCGCT-3´ | **359** |
|  | 5´-CCTACTCGCTGTCCGACTTG-3´ |  |
| ***MAP2*** | 5´-GACATGCAAGGCACAGAAGA-3´ | **251** |
|  | 5´-TTTTCCCTCATGGGAGTCAG-3´ |  |
| ***NURR1*** | 5´-CGATGCCTTGTGTTCAGGCGCAG-3´ | **858** |
|  | 5´-AGCCTTTGCAGCCCTCACAGGTG-3´ |  |
| ***PTX3*** | 5´-GTGGGTGGAGAGGAGAACAA-3´ | **175** |
|  | 5´-TTCCTCCCTCAGGAAACAATG-3´ |  |
| ***LMX1B*** | 5´-GGGATCGGAAACTGTTACTGC-3´ | **218** |
|  | 5´-GTAGTCACCCTTGCACAGCA-3´ |  |
| ***BF1*** | 5´-ACTCAAAACTCGCTGGGCAAC-3´ | **226** |
|  | 5´-CGTGGGGGAAAAAGTAACTGG-3´ |  |
| ***GBX2*** | 5´-CACCACGTCTACGGGCAAGAAC-3´ | **309** |
|  | 5´-AGCTGCTGATGCTGACTTCTGA-3´ |  |
| ***PAX2*** | 5´-ATGTTCGCCTGGGAGATTCG-3´ | **361** |
|  | 5´-GCAAGTGCTTCCGCAAACTG-3´ |  |
| ***PAX7*** | 5´-CCA AGA TTCTTTGCCGCTAC-3´ | **326** |
|  | 5´-CAGGATGCCGTCGATGCTGT-3´ |  |
| ***HOXC8*** | 5´-TTTATGGGGCTCAGCAAGAGG-3´ | **326** |
|  | 5´-TCCACTTCATCCTTCGGTTCTG -3´ |  |
| ***HOXC5*** | 5´-TCGGGGTGCTTCCTTGTAGC-3´ | **290** |
|  | 5´-TTCGTGGCAGGGACTATGGG-3´ |  |
| ***HOXB6*** | 5´-AACTCCACCTTCCCCGTCAC-3´ | **340** |
|  | 5´-CTTCTGTCTCGCCGAACACG-3´ |  |
| ***NKX2.2*** | 5´-TGCCTCTCCTTCTGAACCTTGG -3´ | **337** |
|  | 5´-GCGAAATCTGCCACCAGTTG-3´ |  |
| ***IRX3*** | 5´-AAGAACGCCACCAGGGAGAG -3´ | **473** |
|  | 5´-TTGGAGTCCGAAATGGGTCC-3´ |  |
| ***PAX6*** | 5´-AACAGACACAGCCCTCACAAACA-3´ | **275** |
|  | 5´-CGGGAACTTGAACTGGAACTGAC -3´ |  |
| ***NKX6.1*** | 5´-ACACGAGACCCACTTTTTCCG -3´ | **336** |
|  | 5´-TGCTGGACTTGTGCTTCTTCAAC -3´ |  |
| ***PDGF- R*** | 5'-TCTGCTGGACTGAGAAGTTTCATC-3' | **454** |
|  | 5`-CTATCCACACTGTCAAACAGGTTG-3` |  |
| ***PLP*** | 5'-CTGCTCACCTTCATGATTGC-3' | **343** |
|  | 5’-TGACTTGCAGTTGGGAAGTC-3' |  |
| ***GFAP*** | 5`-TCATCGCTCAGGAGGTCCTT-3` | **383** |
|  | 5`-CTGTTCCCAGAGATGGAGGTT-3` |  |
| ***SOX 10*** | 5`-GCCTGTTCTCCTGGGGCTTTGCTGC-3` | **494** |
|  | 5`-CATCCACCTCACAGATCGCCTACAC-3` |  |
| ***HLXB9*** | 5`- ACCACGCGCA TCCGGGCGCA -3` | **230** |
|  | 5`- CATTTCATCCACCAATTCTG -3` |  |
| ***GAPDH*** | 5`-GTACTCAGCGCCAGCATCG-3` | **302** |
|  | 5`-AGCCACATCGCTCAGACACC-3` |  |
| ***HOXB1*** | 5´-TCAGAAGGAGACGGAGGCTA-3´ | **335** |
|  | 5´-GTGGGGGTGTTAGGTTCTGA-3´ |  |
| ***REX1*** | 5´- AGAATTCGCTTGAGTATTCTGA -3´ | **470** |
|  | 5´- GGCTTTCAGGTTATTTGACTGA -3´ |  |
| ***NCAM*** | 5´- CTTGACCCTGAAGAGCATCC -3´ | **202** |
|  | 5´- GATTGTGGCACTGGGATAGG-3´ |  |
| ***α-FETOPROTEIN*** | 5´-CCATGTACATGAGCACTGTTG-3´ | **358** |
|  | 5´-CTCCAATAACTCCTGGTATCC-3´ |  |
| ***MIXl*** | 5´-GGTACCCCGACATCCACTTGC-3´ | **341** |
|  | 5´-CTCCCATGAGTCCAGCTTTG-3´ |  |
| ***BRACHYURY*** | 5´-CTTCCCTGAGACCCAGTTCAC-3´ | **289** |
|  | 5´-CAGGGTTGGGTACCTGTCAC-3´ |  |
| ***KALLIKREIN*** | 5´-GTTCATGTCAGTGAGAGCTTCCCACC-3´ | **480** |
|  | 5´-TCACATAAGACAGCACTCTGACGGC-3´ |  |
| ***KERATIN 14*** | 5´-ATGATTGGCAGCGTGGAGC-3´ | **390** |
|  | 5´-GTCCAGCTGTGAAGTGCTTG-3´ |  |
| ***HNF-3a*** | 5´-GAGTTTACAGGCTTGTGGCAC-3´ | **390** |
|  | 5´-GAGGGCAATTCCTGAGGATT-3´ |  |
| ***α1-ANTITRYPSIN*** | 5´-AGACCCTTTGAAGTCAAGGACACCG-3´ | **360** |
|  | 5´-CATTGCTGAAGACCTTAGTGATGC-3´ |  |
| ***GATA2*** | 5´-AGCCGGCACCTGTTGTGCAA-3´ | **242** |
|  | 5´-TGACTTCTCCTGCATGCACT-3´ |  |
| ***KERATIN 5*** | 5´-TCAAGGATGCCAGGAACAAG-3´ | **395** |
|  | 5´-GCTTGCACTGAAGCCAGAG-3´ |  |
| ***HIGH SULPHUR KERATIN*** | 5´-AGGAAATCATCTCAGGAGGAAGGGC-3´ | **780** |
|  | 5´-AAAGCACAGATCTTCGGGAGCTACC-3´ |  |
| ***HCG*** | 5´-GTCAACACCACCATCTGTGC-3´ | **285** |
|  | 5´-GGCCTTTGAGGAAGAGGAGT-3´ |  |
| **SNAI1** | 5´-CTCCTCTACTTCAGCCTCTT-3´ | **611** |
|  | 5´-CTTCATCAAAGTCCTGTGGG-3´ |  |
| ***dHAND*** | 5´-AGAAGACCGACGTGAAAGAGGAGA-3´ | **400** |
|  | 5´-ACACGGGAGTGTCCTCTTCGTATT-3´ |  |
| **E-CADHERIN** | 5´-TTCCCTCGACACCCGATTCAAAGT-3´ | **876** |
|  | 5´-AGCTGTTGCTGTTGTGCTTAACCC-3´ |  |
| ***PERIPHERIN*** | 5´-TTGAGTTCCTCAAGAAGCTGCACG-3´ | **605** |
|  | 5´-CACCTCAGGCACAGTCGTCTTTAT-3´ |  |
