# Supplementary figures and images for: Differentiation of Human Embryonic Stem Cells to Regional Specific Neural Precursors in Chemically Defined Medium Conditions
Source: PLoS One. 2008 May 7;3(5):e2122. doi: 10.1371/journal.pone.0002122 (PMC2346555; doi:10.1371/journal.pone.0002122)

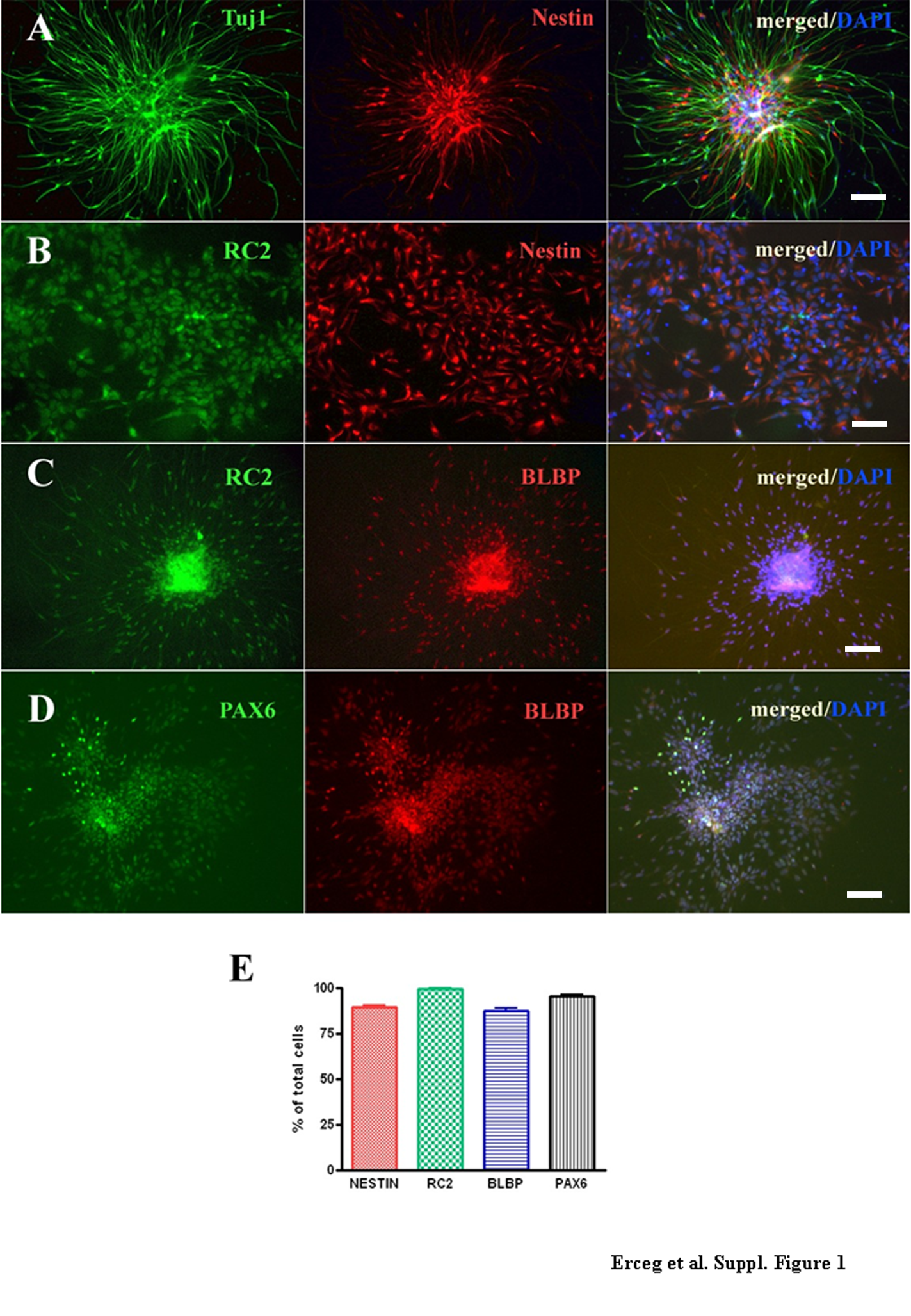

Supplement: Figure S1 — Differentiated cells with radial glia phenotype at D14. (A) The cells are Nestin+ (red coexpressed with Tuj1 and RC2, B). Almost all cells were BLBP+ (red coexpressed with RC2, C and PAX6, D). (E) Percentage of Nestin+, RC2+, PAX6+, and BLBP+ cells. Blue indicates DAPI. Bars: (A–D) 50 µm. Data were averaged and represented as means±S.E.M (E). (10.02 MB TIF) [file pone.0002122.s002.tif]

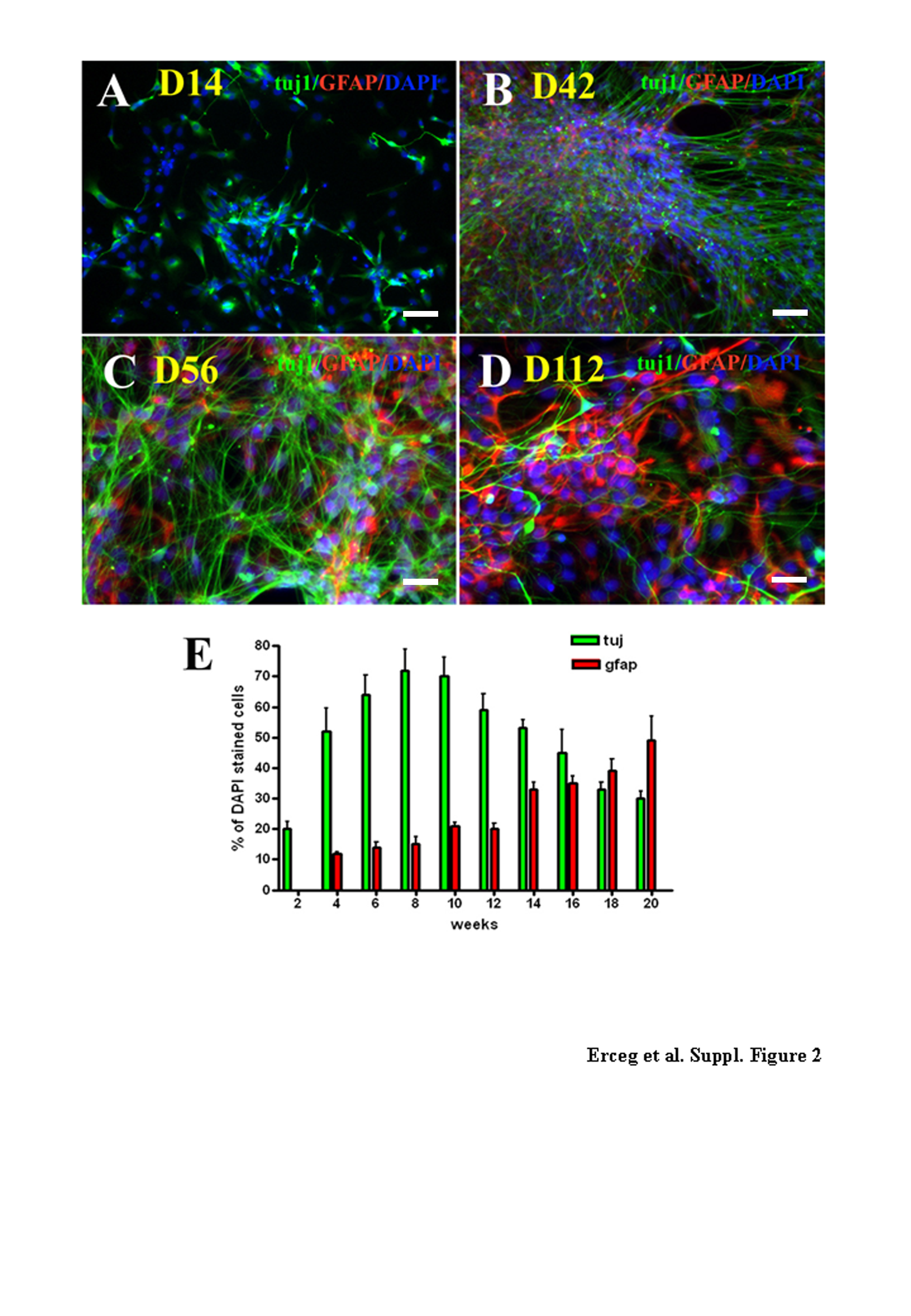

Supplement: Figure S2 — Time-course of gradual neuronal (Tuj1+) to glial (GFAP+) shift. Images of different time-points: D14 (A), D42 (B), D56 (C), D112 (D). The percentage of the cells was analysed by double immunostaining at different time-points of GRM/bFGF or GRM/RA protocols (E). Bars: (A, B) 50 µm; (C, D) 25 µm. Data were averaged and represented as means±S.E.M (E). (10.21 MB TIF) [file pone.0002122.s003.tif]

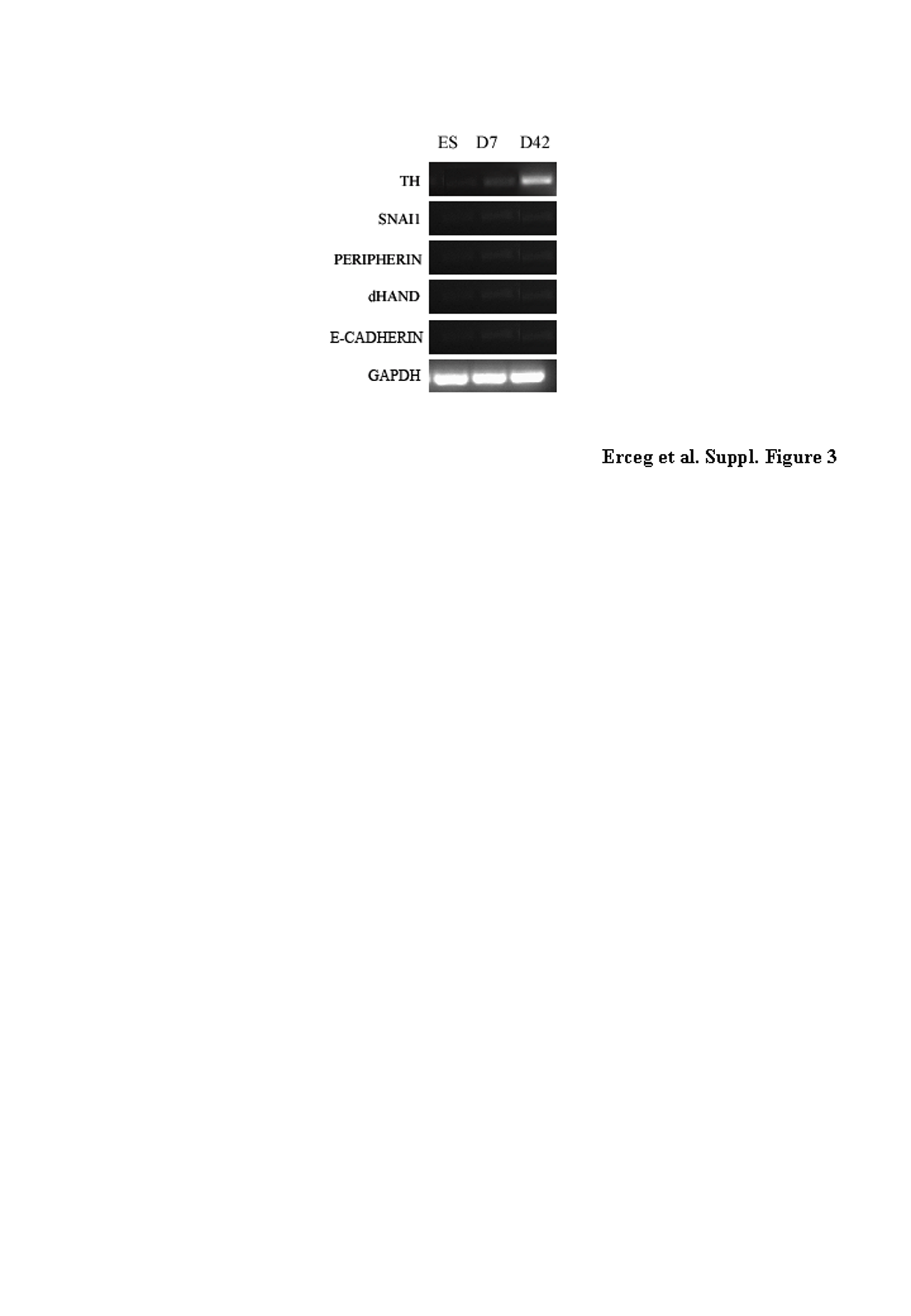

Supplement: Figure S3 — RT-PCR analysis indicates absence of sensory neuron markers. The cells are TH positive, but negative for sensory markers. ES: embryonic stem cells; D7 and D42: day 7 and day 42, respectively. (0.29 MB TIF) [file pone.0002122.s004.tif]
